# Multimodal Magnetic Resonance Imaging Study of Treatment-Naïve Adults with Attention-Deficit/Hyperactivity Disorder

**DOI:** 10.1371/journal.pone.0110199

**Published:** 2014-10-13

**Authors:** Tiffany M. Chaim, Tianhao Zhang, Marcus V. Zanetti, Maria Aparecida da Silva, Mário R. Louzã, Jimit Doshi, Mauricio H. Serpa, Fabio L. S. Duran, Sheila C. Caetano, Christos Davatzikos, Geraldo F. Busatto

**Affiliations:** 1 Laboratory of Psychiatric Neuroimaging, Department and Institute of Psychiatry, Faculty of Medicine, University of São Paulo, Sao Paulo, Sao Paulo, Brazil; 2 Center for Interdisciplinary Research on Applied Neurosciences, University of São Paulo, Sao Paulo, São Paulo, Brazil; 3 Program for Attention Deficit Hyperactivity Disorder, Department and Institute of Psychiatry, Faculty of Medicine, University of São Paulo, Sao Paulo, Sao Paulo, Brazil; 4 Section of Biomedical Image Analysis, Department of Radiology, University of Pennsylvania, Philadelphia, Pennsylvania, Unites States of America; Duke-NUS Graduate Medical School, Singapore

## Abstract

**Background:**

Attention-Deficit/Hiperactivity Disorder (ADHD) is a prevalent disorder, but its neuroanatomical circuitry is still relatively understudied, especially in the adult population. The few morphometric magnetic resonance imaging (MRI) and diffusion tensor imaging (DTI) studies available to date have found heterogeneous results. This may be at least partly attributable to some well-known technical limitations of the conventional voxel-based methods usually employed to analyze such neuroimaging data. Moreover, there is a great paucity of imaging studies of adult ADHD to date that have excluded patients with history of use of stimulant medication.

**Methods:**

A newly validated method named optimally-discriminative voxel-based analysis (ODVBA) was applied to multimodal (structural and DTI) MRI data acquired from 22 treatment-naïve ADHD adults and 19 age- and gender-matched healthy controls (HC).

**Results:**

Regarding DTI data, we found higher fractional anisotropy in ADHD relative to HC encompassing the white matter (WM) of the bilateral superior frontal gyrus, right middle frontal left gyrus, left postcentral gyrus, bilateral cingulate gyrus, bilateral middle temporal gyrus and right superior temporal gyrus; reductions in trace (a measure of diffusivity) in ADHD relative to HC were also found in fronto-striatal-parieto-occipital circuits, including the right superior frontal gyrus and bilateral middle frontal gyrus, right precentral gyrus, left middle occipital gyrus and bilateral cingulate gyrus, as well as the left body and right splenium of the corpus callosum, right superior corona radiata, and right superior longitudinal and fronto-occipital fasciculi. Volumetric abnormalities in ADHD subjects were found only at a trend level of significance, including reduced gray matter (GM) in the right angular gyrus, and increased GM in the right supplementary motor area and superior frontal gyrus.

**Conclusions:**

Our results suggest that adult ADHD is associated with neuroanatomical abnormalities mainly affecting the WM microstructure in fronto-parieto-temporal circuits that have been implicated in cognitive, emotional and visuomotor processes.

## Introduction

Attention-deficit and hyperactivity disorder (ADHD) is a frequent and underdiagnosed mental disorder in the general adult population, with a reported prevalence of up to 5.8% in this age range [1]–[4].

The neuroanatomical circuitry underlying adult ADHD is still relatively understudied. Only a few brain morphometric investigations using magnetic resonance imaging (MRI) have evaluated adult ADHD subjects to date, most of which enrolling patients under pharmacological treatment with stimulant agents. These MRI studies have suggested the presence of gray matter (GM) volume abnormalities in prefrontal-striatal-parietal networks [6]–[8], known to have a central role in attentional processing and executive functioning in general [8]. Other brain regions have also been variably found to present volume abnormalities in adult ADHD patients, including the anterior cingulate cortex, occipital cortex and thalamus [8], [11], [12]. Using diffusion tensor imaging (DTI), MRI studies may also help to unravel the nature of WM microstructural pathological features underlying ADHD in adults, allowing the combined measurement of different indices of white matter microstructural integrity, namely: fractional anisotropy (FA), which provides information on axonal integrity and homogeneity of fiber orientations [13], [14]; and measures of diffusivity, which reflect deficits in cellular density, myelin breakdown or changes in extracellular volumes [13], [15], [16]. The few available DTI studies of adult ADHD subjects to date have reported alterations in tracts that interconnect the above GM regions and which are known to be important for attention and decision making processes, such as the superior longitudinal fasciculus [5], [9], [10].

Thus although not yet numerous, the above MRI studies have provided preliminary evidence that the neuropathology underlying ADHD in adults may involve abnormalities both of GM and WM tracts. However, there has been a great paucity of studies evaluating treatment-naïve adult ADHD subjects to date [17], [18], and no MRI study of such population has acquired both morphometric and DTI data from the same individuals at the same point in time.

Because widely distributed brain networks may be implicated in ADHD, voxel-based methods that systematically search for abnormalities across the entire brain may be more suitable for analyzing data in MRI studies of ADHD, rather than region of interest (ROI)-based methods that are unavoidably restricted to a few selected portions of the brain. However, significant findings have varied considerably across the few MRI studies of ADHD to date that used voxelwise methods, particularly in regard to their specific brain location [7], [19], [20]. Such discrepancies might be, at least in part, attributed to some well-known technical limitations of conventional voxel-based image analysis approaches. For instance, voxel-based morphometry (VBM) methods include a step of image blurring by Gaussian smoothing, in order to account for registration errors and to conform the imaging data to a normal distribution, which is important to validate the assumptions of the statistical parametric mapping approach usually employed in VBM studies [21]. This is conventionally performed in a homogeneous fashion across the whole brain, without accounting for either the wide differences in shape and size of separate brain portions or inter-subject variations; such uniform smoothing methods may increase the risk of both false positive and false negative results [22]. Moreover, the size of the Gaussian kernel is chosen in each study in a somewhat *ad hoc* way, even though it significantly affects the detected group differences. Finally, and most importantly, conventional VBM methods employ a “mass univariate” statistical approach, considering each voxel individually in its separate brain compartment without weighting possible inter-relationships with neighboring voxels and voxels from other brain tissues [23]. For example, in a situation where a specific disorder under study is associated with an enlargement of a given brain area (such as the temporal horn of the lateral ventricle), the VBM analysis may misinterpret such abnormality as a volumetric reduction of a neighboring region (such as the hippocampus). This statistical approach substantially reduces sensitivity to detect group differences and can also produce mislocalization of regions of significant brain tissue density or volumetric changes.

A recently developed voxelwise image analysis methodology, known as optimally-discriminative voxel-based analysis (ODVBA), attempts to minimize the above technical limitations. The ODVBA approach employs a spatially adaptive smoothing filtering and a high-dimensional multivariate local discriminative analysis technique in order to achieve improved sensitivity and accuracy to detect regional brain abnormalities [22]. This methodology has been validated in a recently published study by two of the authors (T.Z and C.D) [24]. In this study, the ODVBA was evaluated using datasets of previously published investigations in schizophrenia and minor cognitive impairment (MCI) in comparison to other established methods, including: the conventional VBM method (Gaussian smoothing plus general linear model – GLM); extant spatially adaptive smoothing methods; and cluster enhancing methods. The experimental results indicated that ODVBA was considerably more sensitive in detecting group differences, demonstrating the highest significance in group differences within the identified voxels. In terms of the spatial extent of detected areas of group differences, agreement of anatomical boundaries and classification, the ODVBA performed better than the other tested voxel-based methods and competitively with the cluster enhancing methods [24]. The findings obtained with the ODBVA approach in those clinical samples were consistent with the previous literature, including widespread GM losses both in schizophrenia and MCI involving several cortical and limbic regions, as well as a greater degree of GM losses in MCI patients who later converted to Alzheimeŕs disease compared to non-converters [24].

In the present MRI study, we employed the ODBVA approach to compare treatment-naïve adults with ADHD and age and gender-matched healthy controls (HC). Given the relevance of assessing both localized morphological abnormalities and microstructural changes in large-scale brain networks associated with ADHD [5], [25], [26], we adopted a multi-modal MRI approach, applying the same voxelwise routines to compare both GM and WM volumes and DTI indices (FA and Trace – TR, an index of diffusivity) between groups. Such combined approach aimed to ascertain whether there are similarities or differences in the topography and severity of GM/WM volumetric versus microstructural WM abnormalities in never-medicated adult ADHD patients. This integrative mapping of morphological and microstructural abnormalities in such population may provide further insights into the neural underpinnings of the clinical manifestations of ADHD in adulthood, including its cognitive and behavioral problems. Also, by combining two DTI indices (FA and TR), we wished to improve understanding about the mechanisms underlying the microstructural WM abnormalities in never-medicated adult ADHD patients. Based on findings from the previous MRI literature cited above [5]–[9], we hypothesized the presence of widespread volumetric abnormalities in the adult ADHD group relative to healthy controls, mainly affecting prefrontal, striatal and parietal regions, as well as microstructural WM abnormalities as assessed with DTI in the tracts which interconnect such brain network, including the caudate nucleus, superior corona radiata, superior longitudinal fasciculus and the cingulum bundle.

## Methods and Materials

### Participants

Twenty-two treatment-naive patients with ADHD aged 18–50 years were consecutively evaluated at the outpatient ADHD clinic of the Institute of Psychiatry, University of São Paulo, Brazil. Subjects were interviewed with the Structured Clinical Interview (SCID) for the DSM-IV [27], and the Schedule for Affective Disorders and Schizophrenia for school-age children-present and lifetime version (K-SADS-PL) [28] Adapted Module (version 6.0) in order to confirm the diagnosis of ADHD and also to access potential axis I comorbidities. For the assessment of symptom severity, we used the Adult ADHD Self-Report Scale (ASRS-18) [29].

Healthy volunteers matched for age and gender with ADHD patients were recruited through advertisement in the local community and constituted our HC group. All subjects in the HC group also underwent clinical interviewing, including the SCID and the K-SADS-PL screening, in order to exclude psychiatric disorders and previous use of psychopharmacological agents.

In addition to the clinical instruments mentioned above, both patients and HC were screened for substance use with the Alcohol Use Disorders Identification Test (AUDIT) [30] and the South Westminster Questionnaire [31]. Diagnostic criteria for substance abuse or dependence were assessed using the SCID [32]. Handedness was assessed using the Edinburgh inventory [33]. Moreover, a general medical history, including information about cerebrovascular risk factors, and data on the use of psychotropic and general medications, was obtained through interviews with patients and/or their family.

Exclusion criteria for both groups were: substance abuse or dependence (current and lifetime); the presence of medical conditions or neurological disorders which could affect the central nervous system; history of mental retardation as assessed by clinical interviews with the patients and a close relative if necessary; past history of head trauma with loss of consciousness; and contraindications for MRI scanning.

This study was approved by our local ethics committee: “*Comissão de Ética para Análise de Projetos de Pesquisa”* – CAPPesq from the board of the University o Sao Paulo Medical School, and “*Comissão Nacional de Ética em Pesquisa – Conep*”. After complete description of the study to the subjects, written informed consent was obtained.

### Image acquisition

All subjects (ADHD patients and HC) underwent MRI scanning using a 1.5T Siemens Espree system (Siemens, Erlagen, Germany).

Morphological data was acquired using a T1-weighted magnetization-prepared rapid gradient echo sequence (MPRAGE) using the following parameters: TR = 2,400 ms, TE = 3.65 ms, NEX = 1, field of view (FOV) = 240 mm, flip angle = 8o, matrix = 192×192 pixels, slice thickness = 1.2 mm (no gap between slices), voxel size = 1.3×1.3×12 mm, resulting in 160 slices covering the whole brain.

The DTI sequence was acquired using cardiac gating, a 12-channel head coil and parallel imaging. DTI was based on an echo-planar image (EPI) acquisition and consisted of one image without diffusion gradient (b = 0 s/mm^2^) plus diffusion-weighted images (DWI) acquired along 64 non-colinear directions (b = 1,000 s/mm^2^) using the following parameters: TR = 8,000 ms, TE = 110 ms, NEX = 2, FOV = 240 mm, matrix = 120×120 pixels, slice thickness = 2.7 mm (no gap between slices), voxel size = 2.0×2.0×2.7 mm, resulting in 50 slices covering the whole brain.

The two sequences were acquired in up to 25 minutes. The imaging protocol also included a T2-weighted turbo spin-echo transaxial sequence (24 slices, slice thickness = 5 mm, 1 mm gap) and a fluid attenuated inversion recovery (FLAIR) transaxial sequence (24 slices, slice thickness = 5 mm, 1 mm gap). Individual image inspection of the datasets of each subject was performed visually by an expert neuroradiologist aiming to identify silent gross brain lesions and artifacts that could interfere with image processing and analysis.

### Processing and analysis of neuroimaging data

The T1-weighted images were pre-processed by correcting for signal inhomogeneities followed by skull-stripping and cerebellum removal. The MNI_N3 Software Package [34], available in: http://www.bic.mni.mcgill.ca/software/N3/, was used for correcting for signal inhomogeneities. Then, the skull-stripping and cerebellum removal were performed using a novel automated method known as Multi-Atlas Skull-Stripping (MASS version 1.0) [35], This method has been validated on three different public data sets and has been shown to achieve a higher accuracy than other state-of-the-art brain extraction methods [35]. It uses a set of selected atlases that are built from the actual dataset under study, based on a classification process that identifies a subgroup of images that best represent the neuroanatomical variations within the sample. Finally, each resulting image was subsequently inspected for imperfections in the skull-stripping and cerebellum removal process (cortical erosions or extra-cerebral tissue remains, for example) and corrections were eventually made manually using the Rordeńs MRIcro 6/2013 program, available in: http://www.mccauslandcenter.sc.edu/mricro/mricron/index.html. As the segmentation algorithms used to extract the GM and WM have been shown to be inaccurate in the cerebellum, due to reduced contrast, our group routinely performs the cerebellum removal. Moreover, the removal of the cerebellum improves our ability to accurately segment GM/WM throughout the brain.

The images were subsequently segmented into their 3 principal brain tissues (GM, WM, and cerebrospinal fluid space) through an automated, iterative algorithm for energy minimization called Multiplicative Intrinsic Component Optimization (MICO) [36], available in http://www.rad.upenn.edu/sbia/software/request.php?software=mico. The skull-stripped and cerebellum-removed images were then spatially registered to the single-subject brain template of Montreal Neurological Institute (MNI) through a robust method for elastic registration called Deformable Registration via Attribute Matching and Mutual-Saliency weighting (DRAMMS version 1.1.0) [37], available in http://www.rad.upenn.edu/sbia/software/dramms/download.html. The deformation field resulting from this spatial registration was then applied to the segmented images in order to generate mass-preserved volumetric maps, named Regional Analysis of Volumes Examined in Normalized Space (RAVENS) maps of the GM, WM, and cerebrospinal fluid compartments [38]. In RAVENS maps, the tissue density reflects the amount of tissue present in each subject’s image at a given location, after mapping to the standardized template space. For example, a region of decreased density indicates a reduced volume in this structure. Lastly, the RAVENS maps were corrected for the total brain volume (given by the sum of all voxels of brain tissue and cerebrospinal fluid space).

The diffusion tensor images were reconstructed from the DWI data using multivariate linear fitting [39]. Spatial normalization of all the tensor images was then carried out via an elastic registration method known as FNIRT [40], against a standard DTI template known as the EVE [41]. For the brain DTI registration we used the FMRIB’s nonlinear image registration tool, which is part of the FMRIB Software Library (FSL 4.1.5) [40], available in http://fsl.fmrib.ox.ac.uk/fsl/fslwiki/FslInstallation. Following the spatial normalization, Fractional Anisotropy (FA) and Trace (TR) images were derived for each subject. While FA provides a measure of diffusion directional anisotropy, TR affords indices of diffusivity, similarly to other commonly employed DTI measures such as mean diffusivity (MD) [42]. TR measures mean diffusivity by adding the Eigen values of the tensor. Thus, it provides a rotationally invariant index of the overall amount of diffusivity within each image voxel [42]. Whereas the TR index provides an estimate of the displacement of water molecules in a medium, measure of FA reflect the degree of water diffusion directionality and, thus, are sensitive to the degree of myelination, density and organization of WM tracts, all of which may reflect maturational processes [22], [42], [43]. One final point to consider is that although the FA and TR indices are thought to reflect *micro*-structural brain abnormalities, the relatively large voxel size of the DTI acquisition (2.0*2.0*2.7 mm) implies that FA differences between groups may not always indicate a *micro*-structural change.

Statistical analyses were performed voxel-by-voxel using the Optimally-Discriminative Voxel-Based Analysis (ODVBA 2.0) Software Package [22], in-house program in SBIA, UPenn, on four different measures: 1) RAVENS maps of GM; 2) RAVENS maps of WM; 3) FA; and 4) TR. ODVBA is a new optimally-discriminative framework for determining the adaptive smoothing kernel and examining group differences. The main premise of ODVBA is that it using a machine learning paradigm to effectively apply a form of matched filtering, to optimally detect a group difference. The framework of ODVBA mainly contains three phases: regional nonnegative discriminative projection, determining each voxel’s statistic, and permutation tests. At first, regional discriminative analysis, restricted by appropriate nonnegativity constraints, is applied to a spatial neighborhood around each voxel, aiming to find the coefficients that best highlight the difference between two groups in that neighborhood. Secondly, since each voxel belongs to a large number of such neighborhoods, each centered on one of its neighboring voxels, the group difference at each voxel is determined by a composition of contributions from all neighborhoods to which it participates. Finally, the statistical significances (*p* values) are obtained by using permutation tests [44]. In this paper, the number of permutations is 2,000.

For the detection of differences in morphometry, anisotropy and diffusivity measures between adult ADHD and HC, we used a strict statistical threshold of p<0.05 with false-discovering rate (FDR) correction for multiple comparisons. Findings that were significant only at an uncorrected threshold of p<0.001 are reported as trends. The significance maps are partitioned and analyzed according to 1) the automated anatomical labeling (AAL) atlas [45] for GM analysis, and 2) the JHU-MNI-ss atlas (or EVE atlas) [46], for WM, FA, and TR analysis. On each anatomical region, we calculated the cluster size and the t statistic (based on the means of the detected area per region).

In addition to the above described between-group comparisons, we conducted a voxel-based search for significant linear correlations between the severity of symptoms as assessed by the ASRS-18 within the ADHD group and each of the four maps (GM, WM, FA and TR), in a regression analysis using the general linear model (GLM) [47].

## Results

### Demographic and Clinical Data

Table 1 summarizes the clinical and demographic characteristics of ADHD patients and HC. All ADHD patients had a history of clinically relevant symptoms starting before the age of 7 years, as assessed with the K-SADS-PL.

**Table 1:** Demographic and clinical characteristics of subjects with ADHD and HC.

Variables	ADHD (n = 22)	HC (n = 19)	Statistical test
Mean age, years (SD)	28.8 (4.9)	28.7 (5.4)	*-*
Gender, no. of males (%)	14 (63.6%)	12 (63.1%)	*-*
Years of Education (mean; SD)	13.8 (2.5)	12.3 (3.8)	F = 0.98, *P* = 0.33, df = 39
Handedness, number of right-handed (%)	20 (90.9%)	18 (94.7%)	χ^2^ = 0.220, df = 1, p = 0.639
Mean ASRS-18 Score (SD)			
Part A: Inattentive Symptoms	30.8 (2.8)	**-**	**-**
Part B: Hyperactive/Impulsive Symptoms	25.9 (5.5)	**-**	**-**

ADHD: Attention-Deficit/Hyperactivity Disorder; HC: Healthy Controls; SD: Standard Deviation; ASRS-18: Adult ADHD Self-Report Scale.

As mentioned above, both groups were matched for age and gender, and all participants had no current of past history of substance use disorders. ADHD patients and HC did not differ with regard to years of education and handedness.

From the 22 patients with ADHD, 10 had the combined subtype whereas 12 presented the inattentive subtype of the disorder. Also, 7 out of the 22 ADHD patients fulfilled criteria for comorbid axis I disorders: 4 for bipolar disorder, 2 for major depressive disorder and 1 for anxiety disorder NOS.

### Multimodal Image Analysis

Significant group differences (at the corrected p<0.05 level) and/or trends (p<0.001 uncorrected) were detected in four comparisons between ADHD patients and HC: 1) GM of ADHD < GM of HC; 2) GM of ADHD > GM of HC; 3) FA of ADHD > FA of HC; 4) TR of ADHD < TR of HC. These differences are detailed in the sub-items below.

### GM of ADHD < GM of HC

**Figure 1:**
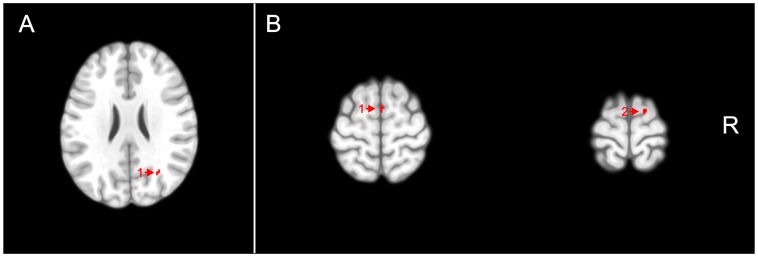
Voxel clusters of relatively reduced gray matter (A) and increased gray matter (B) in attention-deficit/hyperactivity disorder subjects compared to healthy controls are shown in red, overlaid on transaxial sections from a reference brain spatially normalized to the Montreal Neurological Institute stereotactic space, in neurological convention. Statistical maps are displayed with a statistical threshold of *p*<0.001, uncorrected for multiple comparisons. A)1) Angular gyrus (Right); B)1) Supplementary motor area (right); B)2) Superior frontal gyrus (right). R: Right.

There were foci of lower GM values in ADHD patients compared to HC, but these were significant only at the trend level of *p*<0.001, uncorrected for multiple comparisons. These foci were located mainly in the parietal cortex (right angular gyrus), as presented in Table 2. Figure 1 shows the representative slices in the sagittal view.

**Table 2:** Comparison of GM volumes between ADHD and HC.

		Talairach Coordinates*			*p<0.05 (FDR)*		*p*<0.001		*p*<0.001(nonCom)	
Group comparison	Anatomical Regions (Hemisphere)	*x*	*y*	*z*	*N*	*t*	*N*	*t*	*N*	*t*
ADHD < HC	Angular gyrus (right)	41.58	−51.21	24.67	/	/	12	6.68	\	\
ADHD > HC	Supplementary motor area (right)	7.92	14.75	61.9	/	/	21	4.93	24	5.30
	Superior frontal gyrus (right)	15.84	18.44	58.03	/	/	12	6.19	\	\

GM: Gray Matter; ADHD: Attention-Deficit/Hyperactivity Disorder; HC: Healthy Controls; FDR: False Discovery Rate; nonCom: comparison excluding the comorbidities AHD patients; *N*: number of significant voxels in each anatomical region; *t*: value calculated based on the means of Regional Analysis of Volumes Examined in Normalized Space (RAVENS) values of the significant voxels.

*Talairach coordinates represent center-of-mass obtained with the significance level of *p*<0.001.

### GM of ADHD > GM of HC

There were also foci of higher GM values in ADHD patients compared to HC that achieved trend levels of statistical significance (*p*<0.001, uncorrected). These foci of increased GM values in the ADHD group were detected mainly in the right supplementary motor area right superior frontal gyrus. (Table 2, Figure 1).

### FA of ADHD > FA of HC

**Figure 2:**
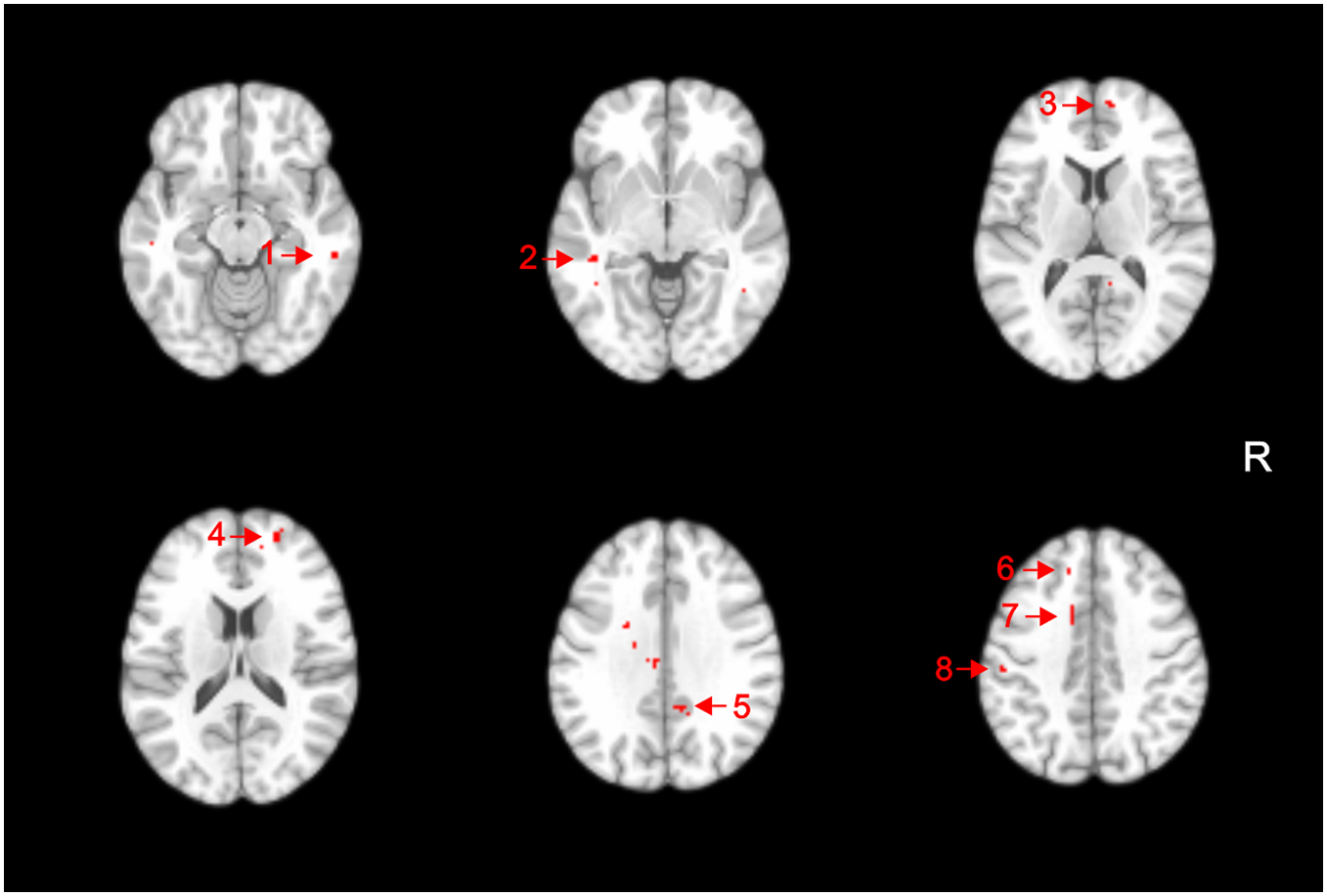
Voxel clusters of relatively increased fractional anisotropy in attention-deficit/hyperactivity disorder subjects compared to healthy controls are shown in red, overlaid on transaxial sections from a reference brain spatially normalized to the Montreal Neurological Institute stereotactic space, in neurological convention. Statistical maps are displayed with a statistical threshold of p<0.05, corrected for multiple comparisons (false-discovery rate). 1) Middle temporal gyrus white matter (WM) (right); 2) Middle temporal gyrus WM (left); 3) Superior frontal gyrus WM (right); 4) Middle frontal gyrus (right); 5) Cingulate gyrus (right); 6) Superior frontal gyrus (left); 7) Cingulate gyrus (left); 8) Postcentral gyrus (left). R: right.

Several areas of significantly increased FA values in the ADHD sample compared with HC were detected at the strict threshold of *p*<0.05 (corrected for multiple comparisons), in WM underlying the bilateral superior frontal gyrus, right middle frontal gyrus, bilateral cingulate gyrus, bilateral middle temporal gyrus and left postcentral gyrus (Table 3, Figure 2). There were additional foci of increased FA values in ADHD patients that were significant at the trend level of p<0.001 (uncorrected), located in WM regions underlying the right superior temporal gyrus and right precentral gyrus, as well as the left posterior thalamic radiation, left inferior longitudinal/left inferior fronto-occipital fasciculi (sagital stratum), left body of the corpus callosum, and left superior corona radiata (Table 3).

**Table 3:** Statistics obtained from the analysis of FA on ADHD >HC.

	Talairach Coordinates*			*p*<0.05 (FDR)		*p*<0.001		*p*<0.001 nonCom	
Anatomical Regions (Hemisphere)	*x*	*y*	*z*	*N*	*t*	*N*	*t*	*N*	*t*
Superior frontal gyrus (right)	11.88	59.04	15.46	40	6.93	85	9.3247	31	6.49
Superior frontal gyrus (left)	−7.92	58.49	4.44	28	5.97	65	8.1677		
Cingulate gyrus (right)	11.88	−48.35	42.95	22	6.38	50	7.1731	28	6.04
Cingulate gyrus (left)	−3.96	−19.38	39.65	21	5.61	35	6.3222	\	\
Middle temporal gyrus (right)	59.4	−31	1.55	18	5.25	33	5.0418	\	\
Postcentral gyrus (left)	−33.66	−33.96	58.81	15	3.67	31	4.6798	101	4.49
Middle frontal gyrus (right)	27.72	57.84	30.26	16	3.94	31	5.064	\	\
Middle temporal gyrus (left)	−41.58	−36.44	9.19	19	5.44	27	5.9271	\	\
Superior corona radiata (left)	−21.78	−2.03	36.94	\	\	48	3.9058	\	\
Body of corpus callosum (left)	−13.86	−7.75	39.07	\	\	18	4.8708	\	\
Superior temporal gyrus (right)	47.52	−38.01	16.64	\	\	17	3.0803	\	\
Precentral gyrus (right)	25.74	−7.11	51.94	\	\	15	8.5404	\	\
Posterior thalamic radiation (left)	−31.68	−60.89	25.15	\	\	14	5.8836	\	\
Inferior longitudinal and Inferior fronto-occipitalfasciculi (Sagital stratum) (left)	−39.6	−34.69	5.42	\	\	11	4.757	\	\

FA: Fractional Anisotropy; ADHD: Attention-Deficit/Hyperactivity Disorder; HC: Healthy Controls; FDR: False Discovery Rate; nonCom: comparison excluding the comorbidities AHD patients; *N*: number of significant voxels in each anatomical region; *t*: value calculated based on the means of fractional anisotropy (FA) values of the significant voxels; WM: white matter.

*Talairach coordinates represent center-of-mass obtained with the significance level of *p*<0.001

### TR of ADHD < TR of HC

**Figure 3:**
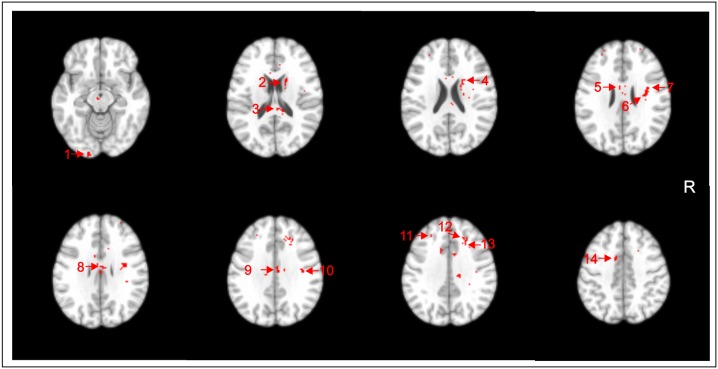
Voxel clusters of relatively decreased Trace in attention-deficit/hyperactivity disorder subjects compared to healthy controls (HC) are shown in red, overlaid on transaxial sections from a reference brain spatially normalized to the Montreal Neurological Institute stereotactic space, in neurological convention. Statistical maps are displayed with a statistical threshold of p<0.05, corrected for multiple comparisons (false-discovery rate). 1) Middle occipital gyrus white matter (WM) (left); 2) Caudate nucleus (right); 3) Splenium of corpus callosum (right); 4) Superior fronto-occipital fasciculus (right); 5) Body of the corpus callosum WM (left); 6) Superior corona radiata (right); 7) Superior longitudinal fasciculus (right); 8) Body of the corpus callosum (right); 9) Cingulate gyrus WM (right); 10) Precentral gyrus WM (right); 11) Middle frontal gyrus WM (left); 12) Superior frontal gyrus WM (right); 13) Middle frontal gyrus WM (right); 14) Cingulate gyrus WM (left). R: right.

Several areas of significantly lower TR values in ADHD subjects compared with HC were seen at the corrected *p*<0.05 level. These were detected in WM regions underlying the right superior frontal gyrus, bilateral middle frontal gyrus, right precentral gyrus, left middle occipital gyrus and bilateral cingulate gyrus, as well as the bilateral body and right splenium of the corpus callosum, right superior corona radiata, right superior longitudinal fasciculus, right superior fronto-occipital fasciculus, and right caudate nucleus (Table 4, Figure 3). There were additional foci of lower TR values in ADHD patients that were significant at the trend level of p<0.001 (uncorrected), located in WM regions underlying the right angular gyrus as well as the bilateral thalamus and left genu of the corpus callosum (Table 4).

**Table 4:** Statistics obtained from the analysis of TR on ADHD < HC.

	Talairach Coordinates*			*p*<0.05 (FDR)		*p*<0.001		*p*<0.001 nonCom	
Anatomical Regions (Hemisphere)	*x* *	*y* *	*z* *	*N*	*t*	*N*	*t*	*N*	*t*
Superior frontal gyrus WM (right)	25.74	33.11	40.71	25	4.7117	62	5.1361	\	\
Middle frontal gyrus WM (right)	27.72	27.21	39.16	16	5.1947	55	6.177	\	\
Precentral gyrus WM (right)	37.62	3.97	37.04	26	5.2873	55	6.0633	\	\
Splenium of corpus callosum WM (right)	9.9	33.49	29.31	29	4.6834	54	4.9279	23	5.1769
Cingulate gyrus WM (right)	3.96	13.66	37.52	27	4.2316	53	4.4117	11	3.7164
Caudate Nucleus (right)	17.82	7.1	25.43	27	3.0545	44	2.9972	\	\
Body of corpus callosum (right)	3.96	9.87	35.49	21	5.5169	42	4.9758	\	\
Cingulate gyrus WM (left)	7.92	8.11	45.65	23	5.48	39	5.5251	\	\
Middle frontal gyrus WM (left)	27.72	55.26	17.49	20	7.571	39	8.8648	\	\
Superior longitudinal fasciculus (right)	35.64	9.78	37.33	26	6.3192	35	7.0118	\	\
Body of corpus callosum (left)	5.94	5.62	34.72	12	5.3907	32	5.2589	\	\
Superior corona radiata (right)	23.76	9.22	29.01	17	7.1591	28	6.1386	\	\
Middle occipital gyrus WM (left)	17.82	104.53	7.07	15	3.7411	24	3.7598	\	\
Superior fronto-occipital fasciculus (right)	19.8	3.4	29.31	15	3.771	16	3.7668	\	\
Thalamus (right)	3.96	9.13	11.51	\	\	18	4.2025	\	\
Angular gyrus WM (right)	45.54	65.7	45.66	\	\	16	6.2677	\	\
Thalamus (left)	1.98	14.85	13.63	\	\	15	3.0199	\	\
Genu of corpus callosum (left)	5.94	26.1	17.11	\	\	14	5.1061	\	\

TR: Trace; ADHD: Attention-Deficit/Hyperactivity Disorder; HC: Healthy Control; FDR: False Discovery Rate; nonCom: comparison excluding the comorbidities AHD patients; *N*: number of significant voxels in each anatomical region; *t*: value calculated based on the means of trace (TR) values of the significant voxels; WM: white matter.

*Talairach coordinates represent center-of-mass obtained with the significance level of *p*<0.001.

### Other group comparisons

There were no statistically significant differences (or trends) between groups when we compared: 1) WM of ADHD < WM of HC; 2) WM of ADHD > WM of HC; 3) FA of ADHD < FA of HC; and 4) TR of ADHD > TR of HC.

### Comparisons between ADHD patients with no comorbidities (n = 15) versus HC (n = 19)

None of the findings reported in the above comparisons retained statistical significance after correction for multiple comparisons (p<0.05). However, the following results remained as trends (p<0.001, uncorrected) (see Tables 2, 3 and 4): 1) GM of ADHD > GM of HC: right supplementary motor area; 2) FA of ADHD > FA of HC: WM underlying the right superior frontal gyrus, right cingulate gyrus, and left postcentral gyrus; and 3) TR of ADHD < TR of HC: right splenium of the corpus callosum and WM underlying the right cingulate gyrus.

### Correlations between ADHD symptom severity and brain imaging measures

The investigation of correlations between ASRS-18 scores within the ADHD group and brain imaging measures revealed no significant results at p<0.05 (corrected for multiple comparisons).

## Discussion

This MRI study investigated brain morphometric and DTI differences between adults with ADHD and age- and gender-matched healthy subjects. In contrast with the vast majority of imaging studies of adult ADHD patients conducted to date, we were able to investigate a group of adult ADHD subjects who had never been exposed to treatment. Samples of treatment-naïve adults with ADHD provide information of critical relevance on brain abnormalities which are genuine to the disease and cannot be attributed to medication effects [26], [62]–[64]. In addition, we employed ODVBA, a newly-validated image analysis methodology aimed at increasing: sensitivity to detect group differences; and accuracy in the localization of brain regions of significant volumetric and WM integrity changes [22]. By evaluating concurrently brain morphology and WM connectivity in the same sample of adult, treatment-naïve ADHD patients, we were able to detect macro- and *micro*-structural changes in a whole-brain fashion, thus potentially affording a more thorough understanding of the network of anatomical abnormalities underlying the clinical manifestations of ADHD [5], [48].

### GM Volume Abnormalities

The present results suggest that adult ADHD is associated with subtle GM volume abnormalities affecting fronto-parietal networks, since there was a statistical trend towards GM reductions in the right angular gyri in our ADHD sample compared to controls, as well as GM volume increases in the right superior frontal gyrus, and right supplementary motor area. These cortical areas are interconnected and participate critically in a number of cognitive processes of relevance to ADHD such as attention, executive functioning and visuomotor coordination [49], [50]. Dysfunction in this circuitry has been linked to behavioral symptoms that are prominent in ADHD, such as altered response inhibition and difficulties with waiting situations [51].

Regions of GM reduction affecting the parietal cortex in adults with ADHD were also found by Makris et al. (2007), who observed a decrease in thickness in the inferior parietal lobule of the right hemisphere [52]. Also, a recent study evaluating a cohort of adults with childhood-onset ADHD found reduced cortical thickness in similar parietal areas, known as the dorsal attentional network [8]. The angular gyrus lies in the caudal inferior portion of the parietal lobe and brings to the prefrontal cortex visual and linguistic information [53]. Likewise, the prefrontal cortex via bidirectional connections sends to the posterior parietal region information for the adjustment of the focus of attention in different parts of the space [5]. The angular gyrus, frequently associated with semantic aspects of language processing [53], has also been implicated in impulsive personality traits [54].

ADHD in children is known to be linked to a retarded cortical maturation relative to normal development [55]–[57]. In children with ADHD, both a delay in attaining peak cortical thickness throughout most of the brain [56] and a slower rate of cortical thinning affecting mainly prefrontal regions, including the premotor area [57], have been observed relative to normal controls. Thus, the trend towards increased GM in frontal regions observed in the adults with ADHD here might be a residue of to abnormal neurodevelopmental processes that occur since childhood [58].

Despite the improved sensitivity of the ODBVA methodology employed herein [24], we found modest GM deficits in adult ADHD subjects relative to controls. The subtlety of our findings of volumetric changes are consistent with the results of previous morphometric MRI studies evaluating GM differences between treatment naïve adult ADHD and HC samples of comparable size to ours, which have reported circumscribed GM deficits in ADHD samples when using strict statistical thresholds with correction for multiple comparisons [59], [60], [61]. It should also be noted that the vast majority of previous imaging studies in ADHD have been conducted with medicated patients. The use of stimulant medication may be associated with changes in regional brain volumes and/or cortical thickness affecting the anterior cingulate cortex, dorsolateral frontal cortex, parieto-occipital region and striatum [26], [62]–[64].

### WM Morphometric and DTI Analyses

No significant focus of WM volume abnormality was observed in the ADHD group relative to controls, even when a flexible statistical threshold of uncorrected p<0.001 was used. Few MRI studies investigated the presence of WM morphological changes in ADHD to date. Studies evaluating children and adolescents with ADHD reported an overall reduction of total cerebral WM as well as mostly bilateral reductions in all four lobes compared to healthy subjects [65]–[69]. In one study of ADHD adults, a trend towards an increase in total WM volume has been reported [70].

Conversely, our analyses of DTI data revealed a widespread pattern of microstructural WM differences between the ADHD and HC groups, as measured, respectively, by FA and TR indices, with findings retaining statistical significance after correction for multiple comparisons. These changes in DTI indices in our sample of adults with ADHD relative to HC were located in fronto-striatal-parieto-temporo-occipital circuits, involving WM regions underlying the frontal, parietal, temporal and occipital lobes, as well as the superior corona radiata, caudate nucleus, superior longitudinal and superior fronto-occipital fasciculi, besides portions of the corpus callosum responsible for the inter-hemispheric connection of frontal, parietal and occipital regions (Tables 3 and 4). The findings of DTI abnormalities affecting these wide WM tracts in adult ADHD patients relative to HC provide support to the hypothesis of impaired connection in widespread brain areas in the pathophysiology of ADHD [23].

It is interesting to note that some of the WM tracts implicated in the present study (eg. the superior longitudinal fasciculi and the body of the corpus callosum) are among the tracts that mature most tardive in normal life, with the age of peak FA and minimum TR varying from 30 to 40 years in healthy aging. Conversely, other WM tracts also implicated herein (eg. splenium of the corpus callosum) show an early developing pattern of maturation in normal life, with the age of peak FA and minimum TR varying from 20 to 30 years in healthy aging [71]. DTI abnormalities affecting these WM circuits have been previously reported by studies of adolescent and adult ADHD [5], [9], [23], [72]–[76]. It is intriguing, however, that the direction of FA alterations observed in ADHD patients relative to controls varies considerably across different investigations, with some studies reporting areas of FA increase [74], [77], [78], others observing foci of FA decrease [9], [5], [73], [75] and one paper showing mixed findings [23]. As the age of peak FA and minimum TR of the WM tracts most frequently implicated in ADHD varies from 20 to 40 years in normal aging [71], differences in the age range of the participants enrolled in the above-mentioned studies might at least partially account for their discrepant results. One other potential source of heterogeneity in the literature about ADHD is the wide variation observed in the methodology employed for the analysis of DTI data. Also, it is conceivable that WM tracts interconnecting the anterior cingulate cortex, dorsolateral frontal cortex, parieto-occipital region and striatum would suffer influence from chronic medication usage and, thus, be a major source of bias in DTI studies of ADHD. To our knowledge, no study to date has evaluated the impact of stimulant medication use on DTI indices.

Although findings of increased FA [17], [79] and reduced diffusivity [79] have been previously reported in ADHD samples including adult patients, our study is the first to report increased FA and reduced TR (with results corrected for multiple comparisons) in adult treatment-naïve patients with ADHD. The FA and TR findings observed in our sample of ADHD adults might suggest that, although a delay in the maturation of WM circuits is observed in the brain development of adolescents with ADHD [80], higher FA and lower TR indices are achieved in adulthood in subjects with persistent ADHD symptoms.

Higher values of FA are typically interpreted as reflecting more highly organized fibers oriented in the same direction [81]–[83], and this might be taken as indicative of healthier white matter [16]. However, recent DTI studies of brain disorders such as Williams syndrome, Alzheimeŕs disease and bipolar disorder have suggested that higher FA values may be actually indicative of WM pathology [84]–[88]. Findings of increased FA in such previous MRI studies have been speculated to indicate less neuronal branching and/or abnormally increased density of WM fibers oriented in the same direction [84]–[88]. In brain areas with significant crossing and branching of neuronal fibers, FA values might be relatively lower due to diffusion in multiple directions. In contrast, areas with less fiber crossing/branching could reduce the magnitude of diffusion in secondary and tertiary directions, leading to relatively greater FA values [89]. According to such reasoning, one possible explanation for our findings is that our pattern of higher FA in adult, treatment-naïve ADHD patients could be due to an aberrant reduction in fiber branching in brain regions where that pattern should be present [74], [78], or to an abnormally increased density of fibers in the same orientation.

In regard to the decrement in TR, this measure reflects the rate of water diffusion through the WM, which was reduced in our adult ADHD patients. Besides the hypothesis of abnormally increased fiber density raised above, findings of decreased TR in brain diseases have been previously suggested to reflect processes of atypical myelination [90], [91]. While a reduction in fiber branching would result in higher TR, increased density of fibers and an atypical higher degree of myelination would decrease TR indices. Overall, we thus suggest that the combination of increased FA and decreased TR in fronto-striatal-parietal- temporo-occipital circuits of treatment-naïve adults with ADHD, as shown by our DTI analyses, may indicate that an abnormal increased density of WM fibers oriented in the same direction, less neuronal branching, and/or a higher degree of abnormal myelination in these WM tracts, contribute to the continuing clinical manifestations of ADHD into adulthood. Neuropathological studies investigating WM histopathological changes in the brains of adult ADHD patients would be important to confirm such potential explanations. However, postmortem investigations of such kind have not been carried out to date. Finally, it should be noted that the size of the clusters of increased FA and reduced TR in our study were relatively small, and there are previous studies in modestly-sized samples of ADHD patients that reported findings in the opposite direction (reduced FA and increased diffusivity) [5], [9], [18], [25], [26], [72], [73]. Therefore, caution must be exercized in the interpretation of our DTI results and replication of these findings in larger samples is clearly warranted.

### Integration of findings of gray matter and white matter abnormalities in ADHD patients

Part of the WM tracts in which we found the greater degree of FA and/or TR differences between ADHD patients and HC are known to interconnect the brain regions where we also detected trend GM volume changes in the ADHD group, namely the fronto-striatal-parieto-temporo-occipital networks. Therefore, taking together the different GM and WM measurements evaluated in the present study, the findings obtained suggest that the persistence of ADHD symptoms into adulthood relates to: circumscribed GM volume abnormalities, subtler than those detected in children and adolescent with ADHD and which are thought to reflect delayed maturation processes [56], [57]; and widespread microstructural alterations in the WM tracts connecting these and other GM regions.

We used exactly the same image processing pipelines and statistical inference approach to the analysis of both morphometric and DTI data obtained from the same individuals in a single MRI scanning session. This allowed us to demonstrate that microstructural WM differences from HC are more widespread and significant than macroscopic GM or WM volumetric changes in never-medicated adult ADHD patients. To the best of our knowledge, such prominence of WM microstructural changes in treatment-naïve ADHD adults has not been reported previously in studies combining DTI and brain volumetric measurements. This pattern of results is consistent with the notion that ADHD-related impairments in cortico-subcortical circuits may originate from alterations in microstructural connectivity [76]. If replicated in subsequent studies, our findings could indicate that microstructural WM abnormalities represent a key neuroanatomical feature underlying the persistence of ADHD symptoms into adulthood.

We did not find any significant results in our correlation analysis between brain imaging measures and the severity of ADHD symptoms. This may relate to an increased risk of type II statistical errors, due to the relatively small size of the ADHD sample. Also, the inclusion of ADHD patients with comorbidities may have increased the clinical heterogeneity of our ADHD sample. However, comorbid psychiatric disorders are very frequent in ADHD [92]; a pure never-medicated ADHD group is very difficult to recruit and would not have been representative of the overall adult ADHD population that usually demands clinical management [93]. We attempted to investigate GM and WM abnormalities in the subgroup with “pure” ADHD features compared to HC, but the results were considerably less significant in comparison to the findings of the analyses for the overall ADHD sample. This may have occurred due to loss of statistical power, given the significant proportion of individuals presenting with comorbid psychiatric conditions in the present study (n = 7). However, one alternative explanation of potential clinical relevance is that the presence of comorbidities is related to an accentuation of the brain abnormalities in never-medicated adult ADHD subjects; this would be consistent with the notion that the presence of comorbid diagnoses is associated with greater ADHD severity [94]–[96]. MRI studies with larger ADHD samples including both subjects with “pure” ADHD and ADHD with comorbidities are needed in order to extend the preliminary findings reported herein.

Finally, two additional methodological limitations should be acknowledged. First, regarding to the protocol used for the acquisition of DTI data, we acquired only one reference “B0” (non-diffusion) image. Although this is a standard strategy [17], [18], [72], [73], [77], [86], it has been recently advised that 1/8th to 1/10th of images during DTI acquisitions should be B0 scans, as this provides greater accuracy in the estimation of tensors and FA values [97]–[99]. Also, we chose to report only TR values rather than adding traditionally used diffusivity indices such as radial diffusivity (RD) and axial diffusivity (AD). Although all diffusion parameters are overall sensitive to tissue properties such as myelination, axonal orientation and axonal density, no DTI measure can be taken as more specific to a given property [13], [43]. Recent studies using synthetic models of crossing fibers have shown that pathological changes to the WM microstructure may result in unpredictable changes to AD and RD measurements, unrelated to the underlying original tissue organization, thus suggesting that such diffusivity indices may not always be reliable [100].

In conclusion, the use of the newly-validated ODVBA image analysis methodology in the present multimodal MRI study revealed the presence of widespread microstructural WM changes in tracts connecting distributed cortical regions in treatment-naïve ADHD adults, as well as subtle regional brain volume abnormalities confined to the GM compartment. Further application of such study design in larger samples is warranted to confirm these preliminary findings and to further investigate how they relate to specific ADHD symptom dimensions, associated neuropsychological deficits, treatment response patterns and other aspects of clinical outcome.
